# Coevolutionary dynamics of phenotypic diversity and contingent cooperation

**DOI:** 10.1371/journal.pcbi.1005363

**Published:** 2017-01-31

**Authors:** Te Wu, Long Wang, Feng Fu

**Affiliations:** 1 Center for Complex Systems, Xidian University, Xi’an, China; 2 Department of Applied Mathematics, The Hong Kong Polytechnic University, Hung Hom, Hong Kong, China; 3 Center for Systems and Control, College of Engineering, Peking University, Beijing, China; 4 Department of Mathematics, Dartmouth College, Hanover, New Hampshire, United States of America; 5 Department of Biomedical Data Science, Geisel School of Medicine, Dartmouth College, Hanover, New Hampshire, United States of America; University of California Irvine, UNITED STATES

## Abstract

Phenotypic diversity is considered beneficial to the evolution of contingent cooperation, in which cooperators channel their help preferentially towards others of similar phenotypes. However, it remains largely unclear how phenotypic variation arises in the first place and thus leads to the construction of phenotypic complexity. Here we propose a mathematical model to study the coevolutionary dynamics of phenotypic diversity and contingent cooperation. Unlike previous models, our model does not assume any prescribed level of phenotypic diversity, but rather lets it be an evolvable trait. Each individual expresses one phenotype at a time and only the phenotypes expressed are visible to others. Moreover, individuals can differ in their potential of phenotypic variation, which is characterized by the number of distinct phenotypes they can randomly switch to. Each individual incurs a cost proportional to the number of potentially expressible phenotypes so as to retain phenotypic variation and expression. Our results show that phenotypic diversity coevolves with contingent cooperation under a wide range of conditions and that there exists an optimal level of phenotypic diversity best promoting contingent cooperation. It pays for contingent cooperators to elevate their potential of phenotypic variation, thereby increasing their opportunities of establishing cooperation via novel phenotypes, as these new phenotypes serve as secret tags that are difficult for defector to discover and chase after. We also find that evolved high levels of phenotypic diversity can occasionally collapse due to the invasion of defector mutants, suggesting that cooperation and phenotypic diversity can mutually reinforce each other. Thus, our results provide new insights into better understanding the coevolution of cooperation and phenotypic diversity.

## Author summary

Phenotypic variation is commonly observed from human cells to the intestinal pathogen Salmonella enterica serovar Typhimurium to the wrinkly-spreader morphs. Such phenotypic diversity proves effective in promoting cooperation, or confers survival advantage against unfavorable environmental changes. Prior studies show that interactions based on phenotypic similarity can promote cooperation. Yet in these models, the level of phenotypic diversity is prescribed such that individuals each possess the same number of available phenotypes, and thereby no evolution of phenotypic diversity per se. We here take into consideration important aspects of the diversity of phenotype and contingent cooperation and show that phenotypic diversity coevolves with cooperation under a variety of conditions. Our work provides a potential mechanism for the evolution of cooperation, and individuals, especially cooperators, endowed with diverse phenotypes constitute the backbone in inducing the coevolution.

## Introduction

How to understand the emergence and persistence of cooperation is a key problem in evolutionary biology [1–12], since individuals sticking to cooperation produce benefits to others at a cost to themselves. The Prisoner’s Dilemma game, as an effective paradigm, has been widely employed to characterize and elucidate the issues surrounding the evolution of cooperation [5, 13–16].

In a typical Prisoner’s Dilemma game, two individuals simultaneously decide either to cooperate or to defect. When both cooperate, they each get the reward *R*. When both defect, they each get the punishment *P*. When a cooperator encounters a defector, the former receives the sucker’s payoff *S* while the later the temptation payoff *T*. The payoff parameters satisfy the inequality *T* > *R* > *P* > *S*. It can be easily obtained that the best response for an individual is to always defect no matter what strategy the opponent adopts in one-shot interaction. For iterated interactions, one additional payoff condition often required is 2*R* > *T* + *P*.

Under these conditions, the aggregate payoff of any two interacting individuals arrives at the highest if both have cooperated among the four possible combinations in terms of the two individuals’ strategies. The strategy maximizing an individual’s payoff (defection) and that maximizing the group’s payoff (cooperation) do not coincide, leading to the social dilemma [1, 2, 4]. To surmount this conflict between group interest and self-interest, some mechanisms must be at work to sustain costly cooperative behaviors. These mechanisms include direct reciprocity [17], indirect reciprocity [18], group selection [19], network reciprocity [5], and kin selection [20].

Besides, it is found that cooperation can arise without reciprocity when individuals preferentially donate to partners sufficiently similar to them [21]. This kind of similarity-mediated interaction is the decisive mechanism that promotes conditional helping behavior (also termed as contingent cooperation or more generally in-group favoritism [22]).

In the original model in Ref. [21], each individual has a tag *τ* and a tolerance *Q*. Individuals acting as donors each have a pre-assigned number of potential recipients to interact with. Their donations are just distributed towards those recipients who share sufficiently similar tags within their tolerance threshold *Q*. The maintenance of cooperation is realized by the successive replacement of one cooperative cluster over another, along with the rise and fall of their tolerance levels. Nevertheless, two open questions remain to be addressed: every agent is a potential donator and thus no absolute defectors (namely, who always defect) are present, and the reflective boundary at *Q* = 0 is biased for the emergence of cooperation. In response to these two questions raised in Ref. [23], the authors of Ref. [21] further corroborated in Ref. [24] that similarity can still breed cooperation even if the option of ‘never donate’ (i.e., negative *Q* values) is allowed, provided that mutations are not biased to ‘never donate’ so strongly as assumed in Ref. [23].

Inspired by this work [21], Traulsen *et al.* constructed a minimal model for tag-based cooperation [25]. By discretizing the tags and tolerances, they explored the evolutionary dynamics in the well-mixed population of infinite size. They later extended this model to structured populations [26] and finite populations [27]. Concerning spatial populations, the authors of Ref. [28] have considered the dynamics of tag diversity in the context of Prisoner’s Dilemma game. Simulation results show that tight coupling between tag and behavioral strategy leads to very drastic oscillations of the dynamics, responsible for the rapid loss of tag diversity and thus of the cooperation. Loose coupling does weaken the oscillation of the dynamics, inducing high levels of overall cooperation in the long run. The model was also analyzed mathematically by omitting the rare event in which recombinations of behavioral strategies and of tags occur simultaneously.

A second set of models analyze the evolution of cooperation under selection-mutation dynamics [29–31]. In the pioneering work [29], just two strategies, contingent cooperation and defection, are considered. Each individual has a phenotype. Cooperators only cooperate with these individuals of the same phenotype. In a birth-death event, both strategy and phenotype can mutate separately. Combining the coalescent theory and perturbation theory, the authors gave the very beautiful criteria, $\left(R-P\right)\left(1+\sqrt{3}\right)>T-S$, for cooperation to evolve. Later, Tarnita *et al.* considered the multiplexity of tags; that is, each individual belongs to *n* groups out of *m* groups and derived the conditions under which cooperation can evolve [30]. Very soon they extended the above framework to study the competition of multiple strategies, and derived the succinct condition for a specific strategy to be selected [31]. Mathematical tractability of these models is possible in the limit of weak selection, which leads to the separation of game payoff and structural coefficients, just depending on the strategy mutation rate and the set mutation rate.

In these models [21, 25, 27–30], phenotype, group, and set can be generally regarded as tags. They are visible and evolving features. Moreover, current interactions neither involve memory of past experience, nor depend on whether partners are altruistic towards third-parties. In a broad sense, reputation [32], social influence, social institutions, accents, and even scientific paradigms [33] can also serve as criteria based on which individuals select their partners [21].

Recent experimental studies have demonstrated that even an isogenic population shows high level of diversity in phenotypic traits [34–41]. Individuals can switch between phenotypes adaptively apart from the induction of mutation, when confronted with changing environments and thus unpredictable threat of survival. To face and surmount these challenges, individuals sometimes randomly switch between different phenotypes. To be more accurate, when cooperators cooperate probabilistically, the cooperative act itself as phenotype exhibits diversity [36, 39]. This work [39] has pointed out that under deme-structured environment the phenotype-mediated interaction environments (assortment) are sufficient to evolve cooperation. Not limited to the evolution of cooperation, the phenotype diversity can also determine the competence of cells in Bacillus subtilis [37]. In response to fluctuating environments, cells may tune the switching rates between phenotypes to maximize their fitness [34]. Even the cell’s phenotype is subject to the density of the inducers in the ambient environment [43]. A key point in these studies seems that phenotype diversity by itself can confer a survival advantage.

These prior studies mainly focus on the importance of stochastic phenotypic expression for the viability of organisms [34–41]. The environmental change is weakly affected by, or totally controlled by some factors independent of, the organisms living in the environment. To realize this stochastic expression, organisms are endowed with multiple phenotypes and they can switch between these phenotypes, a phenomenon known as phenotypic variation (or noise) [37, 40]. These studies have mainly dealt with species-environment systems [34–38, 40, 41], while interactions between subpopulations with different phenotypes are largely unconsidered or simply characterized by mutation at a constant rate [34, 36].

Furthermore, although previous studies concerning tag-based cooperation [21–31] have taken into consideration interactions between subpopulations with different phenotypes, these models focus almost exclusively on how phenotypic diversity affects cooperation. Therefore, how such phenotypic diversity emerges in the first place has yet to be fully answered. Our study shall combine individuals’ strategic behavioral interactions, characterized by the two-player Prisoner’s Dilemma game, with evolvable phenotypic diversity and set out to probe the resulting coevolutionary dynamics.

In this paper, we are interested in the key question of how natural selection leads to phenotype diversity when cooperation is contingent on the phenotypic similarity. Here phenotypes are observable and cooperators channel their help only to these of similar phenotypes. Some questions naturally arise: Why does there exist phenotypic variations in the first place? How does natural selection lead to the emergence of multiple distinctive phenotypes that are expressed in the population? Whether does the contingent cooperation coevolve with diverse phenotypes? We will address the question using a coevolutionary model of phenotypic diversity and cooperation. Our present model does not require any prescribed level of phenotypic diversity, but rather lets it be an evolvable trait. We will show that phenotypic diversity and contingent cooperation can coevolve under wide conditions, and moreover, natural selection favors an optimum level of phenotypic diversity.

## Methods

### Population dynamics

Consider a finite asexual population of *N* individuals. Each individual *i* is characterized by a triplet (*G*_*i*_, *S*_*i*_, *K*_*i*_), where *G*_*i*_ is the current phenotype expressed, *S*_*i*_ is the behavioral strategy, and *K*_*i*_ is the total number of potential phenotypes individual *i* can express. The behavioral strategy *S*_*i*_ is further determined by a pair of variables within the unit square, *S*_*i*_ = [*p*_*i*_, *q*_*i*_] ∈ [0, 1]^2^. Here *p*_*i*_ is the probability that *i* cooperates with similar others, and *q*_*i*_ is the probability that *i* cooperates with others of different phenotypes. For simplicity, we will focus our analysis on two discrete strategies, *C* = [1, 0] and *D* = [0, 0]. It is straightforward to extend the behavioral strategy to the full space similarly as in Ref. [64].

**Fig 1:**
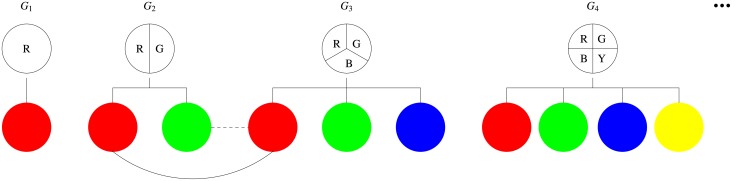
Phenotypic diversity and contingent cooperation. We consider variation in the capacity of expressing different phenotypes. *G*_1_ possesses only one expressible phenotype, say Red. *G*_4_ possesses four expressible phenotypes, say Red, Green, Blue and Yellow. Each individual just expresses one phenotype. *G*_2_ can express either Red or Blue, while *G*_3_ can express Red, Blue or Green. When *G*_2_ and *G*_3_ express the same phenotype, there will be an interaction (solid line) between them. When they express different phenotypes, there will be no interaction between them. The interaction outcome is dependent on their strategic behaviors. When both are cooperators, they each get the benefit *b* − *c*. When both are defectors, they get zero payoff each. When a cooperator meets a defector, the former gets the payoff −*c*, while the later reaps the payoff *b*.

The population is well-mixed, and individuals interact with everyone else. The interactions are characterized by the simplified Prisoner’s Dilemma game (donation game). A (conditional) cooperator pays a cost *c* for a compatible recipient, of the same phenotype, to receive a benefit *b* (see Fig 1). Defectors pay no costs and distribute no benefits. Each individual *i* incurs a cost *κ*_*i*_ for retaining phenotypic variation and expression. *κ*_*i*_ is assumed to be proportional to *K*_*i*_, *κ*_*i*_(*K*_*i*_) = *θK*_*i*_. Here we choose the simplest possible phenotype cost function. The net payoff *π*_*i*_ determines the reproductive success (fitness) *f*_*i*_ of an individual *i*. Here the fitness is an exponential function of payoff *f*_*i*_ = *e*^*βπ*_*i*_^, where *β* is the intensity of selection.

The evolutionary updating occurs according to a frequency-dependent Moran process. At each time step, an individual is chosen with probability proportional to its fitness to reproduce an offspring. Following birth, a random individual in the population dies. The population size is thus constant throughout the evolution. Reproduction is however subject to mutation. With probability *μ*, the offspring randomly adopts one of the two behavioral strategies and also acquires a random number $K_{i}^{{}^{\prime }}$ of potentially expressible phenotypes at a cost $\theta K_{i}^{{}^{\prime }}$. This mutant expresses one phenotype at random out of the total $K_{i}^{{}^{\prime }}$ possible phenotypic variations.

When it comes to phenotypic switching, we consider the random case. Here we have a combinational mutation that can change behavioral strategy and phenotypic diversity.

### Fixation probability

We now briefly elucidate the general procedure for calculating fixation probabilities. Suppose the population consists of *i* type *A* individuals and *N* − *i* type *B* individuals. Each type *A* individual possesses *K*_*A*_ potentially expressible phenotypes and its strategic behavior is *s*_*A*_. Each type *B* individual possesses *K*_*B*_ potentially expressible phenotypes and its strategic behavior is *s*_*B*_. *s*_*A*_ = 1 if *A* is a cooperator, and *s*_*A*_ = 0 otherwise. So does *s*_*B*_. Denote by *ϕ*_*i*_ the fixation probability that the population eventually arrives at the state consisting of *N* type *A* individuals when it starts with the state consisting of *i* type *A* individuals. The updating event is frequency-dependent Moran process. The payoff for an *A* and a *B* can be respectively written as *P*_*A*_ = (*i* − 1)(*b* − *c*)*s*_*A*_ + (*N* − *i*)(*bs*_*B*_ − *cs*_*A*_)*δ* − *θK*_*A*_, and *P*_*B*_ = *i*(*bs*_*A*_ − *cs*_*B*_)*δ* + (*N* − *i* − 1)(*b* − *c*)*s*_*B*_ − *θK*_*B*_, with *δ* being one if *A* and *B* have expressed the same phenotype and zero otherwise. Self-interaction is obviously not included. The fitness for A and B reads *f*_*A*_ = *e*^*βP*_*A*_^, and *g*_*B*_ = *e*^*βP*_*B*_^, respectively. The intensity of selection *β* measures how much payoff contributes to fitness. In an updating event, the probability for the number of type *A* individuals in the population to increase by one, decrease by one and remain unchanged is given respectively as $T_{i,i+1}=\frac{if_{A}}{if_{A}+\left(N-i\right)g_{B}}\cdot \frac{N-i}{N}$, $T_{i,i-1}=\frac{\left(N-i\right)g_{B}}{if_{A}+\left(N-i\right)g_{B}}\cdot \frac{i}{N}$, and *T*_*i*,*i*_ = 1 − *T*_*i*,*i*+1_ − *T*_*i*,*i*−1_. Then we have

$$\phi_{i}=T_{i,i+1}\phi_{i+1}+T_{i,i-1}\phi_{i-1}+T_{i,i}\phi_{i}$$

Combining with the boundary conditions *ϕ*_0_ = 0 and *ϕ*_*N*_ = 1, we can obtain the fixation probability as

$$\phi_{1}={\left(1+\sum_{l=1}^{N-1}\prod_{k=1}^{l}\frac{T_{k,k-1}}{T_{k,k+1}}\right)}^{-1}$$

### Stationary distribution

Individuals possessing too many available phenotypes will be easily invaded by those who are endowed with a modest number of phenotypes for possessing cost increases linearly. In the long run, their fraction is almost negligible. We can thus assume that individuals can be endowed with at most *M* potentially expressible phenotypes. In the limit of small mutation, the population is quite frequently located at one of these 2*M* homogeneous states. This state is from time to time disturbed by the mutant. Very soon either the mutant is wiped out and the homogeneous state is recovered, or it successfully invades and wipes out the residents and thus transits the population to a new homogeneous state. Therefore, the population dynamics of 2*M* strains can be well approximated by an embedded Markov chain between these *M* full defective states and *M* full cooperative states. For convenience’s sake, we label cooperative strains with even numbers 2*K*_*C*_, and defective strains with odd numbers, 2*K*_*D*_ − 1, for 1 ≤ *K*_*C*_ ≤ *M* and 1 ≤ *K*_*D*_ ≤ *M*. For strain *X* having *K*_*X*_ potentially expressible phenotypes and strain *Y* with *K*_*Y*_ potentially expressible phenotypes, the expected transition rate from state *X* to state *Y* is *r*(*X*, *Y*;*K*_*X*_, *K*_*Y*_) as shown by Eq (1) in Results Section. Going a further step, we can get the transition matrix *A* with dimension 2*M* by 2*M*. The *ij*th entry of matrix *A* is *r*(*i*, *j*;*K*_*i*_, *K*_*j*_) for *i* ≠ *j*, and the *ii*th entry is one minus the sum of all other entries in the *i*th row. It should be noted that we have analytically derived the transition rates between any two competing strains and thus the transition matrix. We then use built-in numerical methods in Matlab to solve the left eigenvector of the transition matrix corresponding to the eigenvalue of one. This left eigenvector, after normalization as needed, gives the stationary distribution of these 2*M* full states. Summing all the elements with even indices in the normalized eigenvector, we can get the overall cooperation level [44, 45].

## Results

### Pairwise invasion dynamics

Let us start with the simplest case of two competing strains, which constitutes the basis for analyzing the general population dynamics. One strain has the potential of expressing *K*_*X*_ distinctive phenotypes; the other has the potential of expressing *K*_*Y*_ different phenotypes. Mutations among these two strains are bidirectional and occur at a sufficiently small rate. At this limit of small mutation rates, the competition dynamics can be simplified by investigating transition rates between homogeneous population states (All C vs. All D). Let $\rho_{X\to Y}^{s}$ be the fixation probability that a single mutant *X* takes over the resident population *Y* when *X* and *Y* are of the same phenotype. Let $\rho_{X\to Y}^{d}$ be the fixation probability that a single mutant *X* takes over the resident population *Y* when *X* and *Y* have expressed different phenotypes.

Then the expected transition rate (omitting the mutation rate *μ* for notational brevity) from state *X* to *Y*, *r*(*X*, *Y*;*K*_*X*_, *K*_*Y*_), is given by

$$\begin{array}{ccc} r(X,Y;K_{X},K_{Y}) & = & H\left(K_{Y}-K_{X}\right)\left[\frac{1}{2}\alpha_{Y}\rho_{Y\to X}^{s}+\frac{1}{2}\left(1-\alpha_{Y}\right)\rho_{Y\to X}^{d}\right]\\ & + & \left[1-H\left(K_{Y}-K_{X}\right)\right]\{\frac{1}{2}\frac{K_{X}-K_{Y}}{K_{X}}\rho_{Y\to X}^{d}\\ & + & \frac{1}{2}\frac{K_{Y}}{K_{X}}\left[\alpha_{Y}\rho_{Y\to X}^{s}+\left(1-\alpha_{Y}\right)\rho_{Y\to X}^{d}\right]\}. \end{array}$$ (1)

*H*(⋅) is the Heaviside step function. *H*(*x*) = 1 for *x* ≥ 0, and *H*(*x*) = 0 for *x* < 0. For random phenotypic switching, *α*_*X*_ = 1/*K*_*X*_, where *X* ∈ {*C*, *D*}. It is the same case with *α*_*Y*_. Some explications concerning this transition rate are necessary. When the number of potentially expressible phenotypes that strain *Y* possesses exceeds or equates with that strain *X* possesses, strain *Y* has the chance *α*_*Y*_ to express the same phenotype with strain *X*. At this time, the population moves from state *X* to state *Y* with the probability $\rho_{Y\to X}^{s}$. With probability 1 − *α*_*Y*_, strain *Y* expresses different phenotype from strain *X*. Once this happens, the population moves from state *X* to state *Y* with the probability $\rho_{Y\to X}^{d}$. The coefficient $\frac{1}{2}$ means that the mutant can be either a cooperator or a defector with equal probability. The sum constitutes the first term in the right-hand side of *r*(*X*, *Y*;*K*_*X*_, *K*_*Y*_). Following the same logic, we can arrive at the second term.

We can use Eq (1) to analytically derive the transition rates between different population states in the limit of rare mutations and for any intensity of selection *β*. In particular, Eq (1) can be greatly simplified in the limit of strong selection, *β* → ∞ (see S1 Supplementary Information).

**Fig 2:**
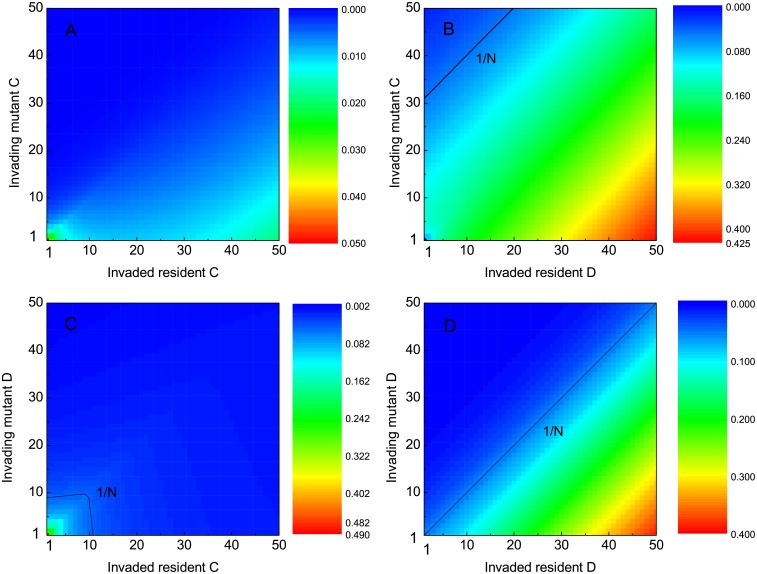
Pairwise invasion plots. Transition rate means the probability that the population moves from an invaded state to an invading state. The capital letter, C or D, along the Y-axis, denotes the mutant’s behavioral strategy, while the one along the X-axis denotes the residents’ behavioral strategy. The coordinate value denotes the number of potentially expressible phenotypes that individuals are endowed with. Parameters: *N* = 20, *b* = 1, *c* = 0.3, *β* = 0.1, *θ* = 0.1.

Fig 2 shows the transition rate with respect to four different strategy combinations of mutants and residents. When a defector attempts to invade the otherwise defector population, the dynamical scenario is easily understandable. Defectors pay no cost and bring no benefit to others. Their fitness is totally dependent on the number of potentially expressible phenotypes. As long as the cost of possessing potentially expressible phenotypes is nonzero, the more potentially expressible phenotypes a defector possesses, the higher cost it bears. Fitness-driven competition puts such defectors in a disadvantageous place, as illustrated in Fig 2D. Actually, we can rigorously confirm this observation. Assume that the invading defectors have *K*_*Y*_ available phenotypes, while the resident defectors are endowed with *K*_*X*_ phenotypes. No matter what the composition of the population is, and independent of their actual expressions, the fitness is *e*^−*βθK*_Y_^ and *e*^−*βθK*_X_^, for an invader and a resident, respectively. After a simple calculation, we can obtain the accurate expressions of the fixation probability as $\rho_{Y\to X}^{s}=\rho_{Y\to X}^{d}=\frac{1-e^{\beta \theta (K_{Y}-K_{X})}}{1-e^{N\beta \theta (K_{Y}-K_{X})}}$, which are also exactly the transition rates for both *K*_*Y*_ > *K*_*X*_ and *K*_*Y*_ < *K*_*X*_. It is very easy to verify that the transition rate is decreasing with respect to *K*_*Y*_, and increasing with respect to *K*_*X*_, respectively. Therefore, a peak appears when the residents are endowed with 50 potentially expressible phenotype and the invaders only with 1.

When the mutants are cooperators and strive to dominate the defector residents, the evolutionary race proceeds in a different way. Not only the cost of phenotypic variation and expression is involved, the payoff resulting from the game interactions is also integral component of fitness. It is better for these cooperators to express different phenotype from the defectors. Thus they can escape from the exploitation of the defectors. In the light of mathematical language, the fixation probability $\rho_{C\to D}^{d}$ is larger than $\rho_{C\to D}^{s}$. We can approximately speak that $\left(1-\alpha_{Y}\right)\rho_{C\to D}^{d}$ is far larger than $\alpha_{Y}\rho_{C\to D}^{s}$ especially for large *K*_*Y*_. It is worth noting that for *K*_*C*_ > *K*_*D*_, $r\left(D,C;K_{D},K_{C}\right)=\frac{1}{2}\alpha_{C}\rho_{C\to D}^{s}+\frac{1}{2}\left(1-\alpha_{C}\right)\rho_{C\to D}^{d}$; for *K*_*C*_ < *K*_*D*_, $r\left(D,C;K_{D},K_{C}\right)=\frac{1}{2}\left(1-\alpha_{D}\right)\rho_{C\to D}^{d}+\frac{1}{2}\alpha_{D}\rho_{C\to D}^{s}$. The analysis bespeaks that the transition rate is mainly controlled by $\rho_{C\to D}^{d}$, and responsible for the similarity of the transition rate relying on *K*_*C*_ and *K*_*D*_ for cases that cooperators invade defectors (see Fig 2B), and that defectors invade defectors. In the former case, the transition rate sees an appreciable increase. This should be attributed to the mutual breed of the invading cooperators once more than one cooperators emerge.

We next probe how the transition rate is dependent on *K*_*C*_ and *K*_*D*_ when defectors attempt to invade the cooperator residents (see Fig 2C). In order to avoid being exploited by the invading defectors, cooperators have no other choice but to increase the number of their potentially expressible phenotypes. In doing so, cooperators need to bear higher cost of phenotypic variation. At the same time, they complicate their own ‘code’, which takes defectors longer time to decipher, thereby allowing contingent cooperators to benefit from their mutual breed for a longer period. As a result, it to some extent reduces the rate that defectors invade cooperators, favoring the persistence of cooperators. These are two driving forces acting on the coevolutionary dynamics. In the given parameter scope (*K*_*C*_ ≤ 50), the positive effect owing to the similarity-mediated interactions predominates. The extent of such predominance is sensitive to the increase in *K*_*C*_. We should bear in mind that once setting the parameters of *b* and *c*, the relative advantage of defectors to cooperators is constant for each interaction. The cost of phenotypic variation nonetheless rises to infinity as *K*_*C*_ goes to infinity. As a consequence, there exists a threshold of *K*_*C*_ above which the reciprocity coming from similarity-mediated interactions is completely offset by the prohibitively high cost of maintaining too many phenotypes. Similar sensitivity on *K*_*C*_ can be observed when cooperators attempt to invade cooperators. Since the invading cooperators are also able to accomplish the reciprocity between themselves, the residents’ strength in resisting invasion is greatly discounted, especially for large *K*_*C*_. This perfectly explains why the transition rate depends on *K*_*C*_ in a *U*-shaped way (see Fig 2A).

### Full population dynamics with 2*M* strains

With the simplified dynamics being scrutinized, it naturally steers us to analyze the full population dynamics. Consider the competition of an arbitrary number of strains (= 2*M*) in the population of finite size (= *N*). As we have pointed out, individuals endowed with very large numbers of available phenotypes are destined to be wiped out only if phenotypic variation is costly. It makes sense to assume *M* as a finite number. In the limit when mutation rarely happens, we are able to analytically compute the average frequency of each of these 2*M* strains in the long run. In this limit, either the initially rare mutants are assimilated by the residents or the mutants successfully invade and take over the whole population before the occurrence of next mutation. Thus there will be at most two different strains present in the population simultaneously. As the transition rate between any two strains has been derived in Eq (1), the embedded Markov chain is well defined. We can thus get the average fraction of time the population spends in each of these 2*M* homogeneous states by computing the stationary distribution of the transition matrix. The stationary distribution is given by the normalized eigenvector of the transition matrix corresponding to its maximal eigenvalue one. Though the approximation is obtained in the limit, it proves valid for a wider range of mutation rate, as we have corroborated by numerical simulations.

**Fig 3:**
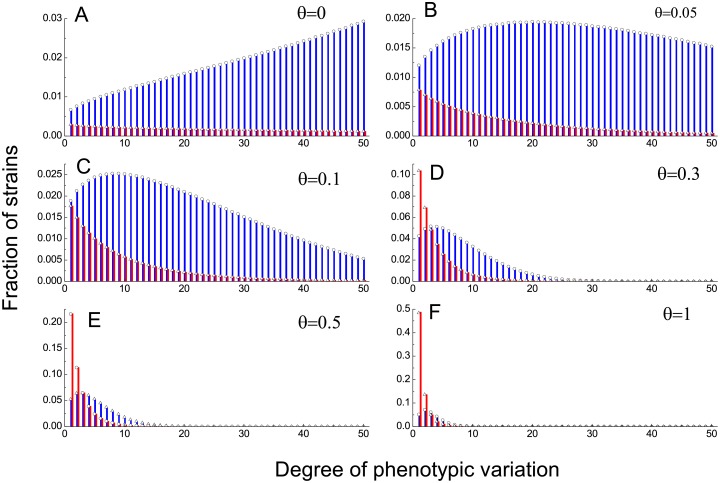
Stationary distribution for 2M competing strains. Fraction of these 2*M* strains in the long run. The bars are obtained by solving the eigenvector of the 2*M* by 2*M* transition matrix. Empty circles and empty triangles are obtained by simulations. Blue denotes cooperator, and red defector. The abscissa value represents how many potentially expressible phenotypes individuals can switch to. The evolutionary process is fully characterized in the main text. Parameters: *N* = 20, *b* = 1, *c* = 0.3, *β* = 0.1, *μ* = 0.002. From A to F, *θ* is 0, 0.05, 0.1, 0.3, 0.5, 1, correspondingly, and the overall cooperation level is 0.91, 0.88, 0.84, 0.63, 0.49, and 0.29, respectively.

Fig 3 illustrates the stationary distribution of these 2*M* strains for different values of parameter *θ*. The abscissa value denotes the number of potentially expressible phenotypes that the corresponding strain can express. As to which phenotype the strain specifically expresses, it depends entirely on the outcome of ‘throwing a dice’ when the strain first emerges as a mutant. Some remarkable features are revealed in this plot. On the one hand, of all cooperative strains there exists an optimal solution $K_{C}^{*}$ in term of phenotypic variation. This optimal number lies in between one and the maximally allowed number and is subject to the change of *θ*. For the cooperative strain possessing this optimal number of potentially expressible phenotypes, it attains the highest fraction among all *M* cooperative strains. For the defective strain, possessing just one potentially expressible phenotype is always the best choice. On the other hand, an increase in *θ* depresses the overall cooperation level, and reduces the diversity as well. For *θ* = 0.05 and 0.1, not only the cooperation level is significantly higher than the defection level, the cooperative strain possessing $K_{C}^{*}$ available phenotypes also accounts for the largest fraction in the long run. As *θ* increases to ∼0.3, cooperation still enjoys appreciable dominance over defection, whereas the defective strain with *K*_*D*_ = 1 is most prevalent. When *θ* is as high as 0.5, the diversity, especially of cooperative strains, drastically shrinks. Concomitantly, the cooperation level drops below 50%. Furthermore, the diversity almost gets lost completely and cooperation level slumps towards zero for extremely high costs such as *θ* = 1. We thus confirm that cooperation does coevolve with the phenotypic diversity. A little differently, for *θ* = 0, the more potentially expressible phenotypes cooperative strain possesses, the more prevalent it is.

**Fig 4:**
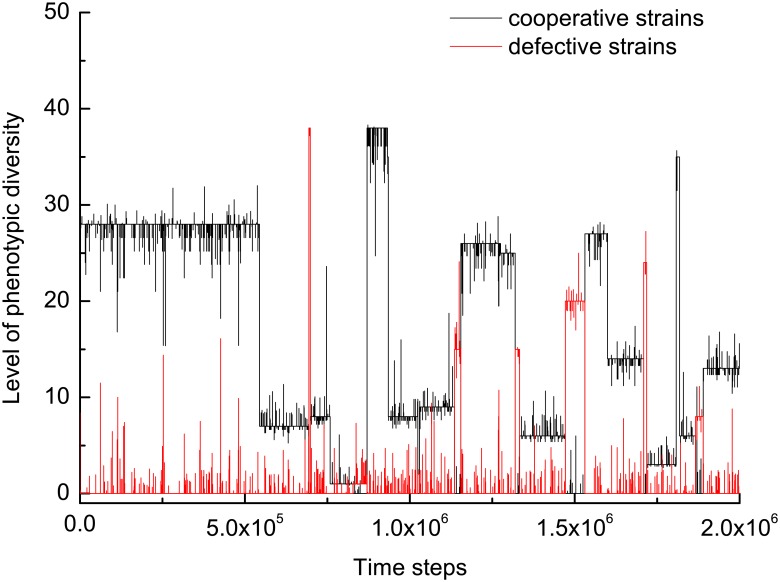
Time evolution of the competition between cooperative strains and defective strains. When the population is occupied by defective strain, it is most likely that the defective strain possesses a very small number of potentially expressible phenotypes. It is either followed by the invasion of defective strain with similar numbers of phenotypes, or by cooperative strain with a moderate number of phenotypes. In the former case, the evolutionary process advances just as it starts. In the later case, it gets very hard for the cooperative strain to be invaded, since it possesses the strongest resistance power against invasion of other strains. In the average sense, cooperative strain endowed with a moderate number of phenotypes prevails most of the time. Parameters: *N* = 20, *b* = 1, *c* = 0.3, *β* = 0.1, *μ* = 0.002, and *θ* = 0.1.

Our findings have demonstrated that there exists an optimal number of phenotypic variations for cooperators and such cooperators account for the highest fraction in the stationary distribution. Even more, such cooperators can be dominated by defectors if the cost of phenotypic variation further increases. However, cooperators still enjoy a higher overall fraction. Reasons responsible for these observations are interesting and fundamental. Fig 4 offers a graphic illustration. For convenience of describing this evolutionary path, we denote by *L*ow, *M*iddle, and *H*igh level when the average phenotype diversity is located in the interval (0, 3], (3, 38], and (38, 50], respectively. Denote by *C*_*L*_ the state of cooperators with *L*ow average phenotype diversity, and the like. When the population is occupied by *C*_*L*_, the dynamics are extremely stable and stay put with a probability as high as 0.99978. The coexistence states, *C*_*L*_ + *D*_*L*_ and *C*_*M*_ + *D*_*L*_, are also visited quite frequently. When *C*_*L*_ + *D*_*L*_ dominates the population, the dynamics are quite unstable. The population either maintains the state with probability 50.55%, or enters into the state *C*_*M*_ with probability 27.4%. When *C*_*M*_ + *D*_*L*_ dominates the population, the dynamics are less stable. The population is as likely as 52.56% to stay in the current state, and also has an odds of 44.22% to enter the state *C*_*M*_ directly. Once the state *C*_*M*_ dominates the population, the dynamics are also extremely stable and stay put with a probability as high as 0.99977. Switching between these four states constitutes the core component of the population dynamics and thus explains the macroscopic observations. It it worth noting that though *D*_*M*_ can stabilize the dynamics with probability 0.99976, paths arriving at this state are so parsimoniously few that this state can produce no essential effects in the long run.

**Fig 5:**
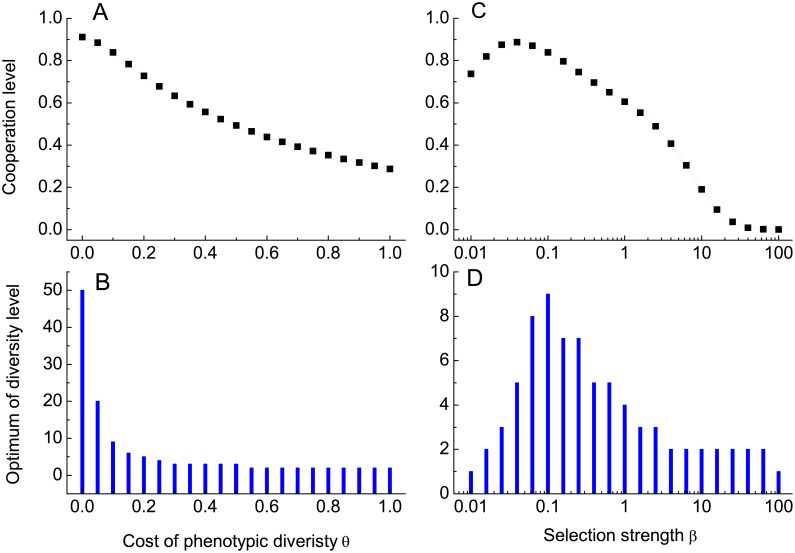
Cooperation level, the optimal phenotypic diversity of cooperative strain as a function of *θ* and *β*, respectively. The overall cooperation level decreases with *θ*. So does the optimal diversity. Even when cooperation is disfavored, the optimal diversity of all cooperative strains still exists. Quite differently, there exists an optimal selection intensity at which the overall cooperation level arrives at the highest and, correspondingly the optimum of diversity level is maximized. Parameters: *N* = 20, *b* = 1, *c* = 0.3. In panels *A* and *B* *β* = 0.1. In panels *C* and *D* *θ* = 0.1.

Following common practice, we investigate how the overall cooperation level and corresponding optimal phenotypic diversity (i.e., $K_{C}^{*}$) vary with the key parameter *θ*, and intensity selection *β* [46–48], respectively. Fig 5*A* shows that the cooperation level monotonically decreases as *θ* rises from 0 to 1. For *θ* being zero, cooperation is stabilized at a level as high as 0.91. Accordingly, the optimal phenotype diversity is the largest allowable number. At this point, complicating phenotype diversity proves more effective in dodging defectors’ exploitation. As long as *θ* is non-zero, the effectiveness is compromised by the cost of having more potentially expressible phenotypes. Consequently for a wide spectrum of *θ*, $K_{C}^{*}$ comes in between 1 and *M*. The higher the cost, the lower $K_{C}^{*}$ is (see Fig 5*B*). These results further corroborate our aforementioned findings. Of interest, Fig 5*C* shows that the cooperation level sees a non-monotonous dependency on the selection intensity *β*. That is, the cooperation level peaks at *β*_*c*_ ≈ 0.0398. Below this value, increasing *β* contributes to the positive effect of cooperative strain clustering relatively far more than the competitive advantage of defective strains over cooperative ones. Once *β* exceeds this threshold *β*_*c*_, *β*’s increment further intensifies the competition, which leaves less space for cooperative strains to escape from defective strains’ stalking and subsequent exploitation. This leads to the decline of cooperation level as *β* rises beyond *β*_*c*_. Similar dependency of the optimal diversity level on *β* can be observed (see Fig 5D). Moreover, although for the extreme values of *β* (0 and ∞) the optimal diversity does not exist, the underlying rationales are different. For the former case, evolution of cooperation follows the neutral drift. All strains account for equal fraction in the long run. For the later case, the defective strain with just one potentially expressible phenotype overwhelmingly predominates the evolutionary race, leaving negligible odds for other strains to prevail.

## Discussion

How altruistic behavior emerges and persists is a key issue to be answered [49–57]. The results of our model provide a possible path for cooperation to get established, as the evolving phenotypic diversity plays a crucial role [58–61]. As far as defective strains are concerned, competition weakens the survivability of the ones endowed with large numbers of potentially expressible phenotypes, thereby suppressing the phenotypic diversity of defective strains. It is the other way around for cooperative strains. In addition to the strain with the optimum level of phenotypic diversity, $K_{C}^{*}$, those endowed with the number of potentially expressible phenotypes close to $K_{C}^{*}$ almost share the same strong ability to withstand the invasion of other strains. It follows that all these cooperative strains account for commensurate fractions, and as a consequence, the phenotypic diversity of cooperative strains is preserved. Thanks to the positive effect of phenotypic diversity on cooperation, the aggregate competence of cooperative strains can readily outperform that of defective strains, leading to the overall cooperation level higher than 50%. It is noteworthy that as *θ* increases to 0.3, the fraction of the most prevalent defective strain is higher than that of the cooperative strain with $K_{C}^{*}$. Even so, the overall cooperation level is still over 0.5, suggesting the vital role of diversity in the establishment of cooperation once again.

For very large *θ*, contribution to fitness generated by game interactions is incommensurate to that of the cost of phenotypic variation and expression. In other words, the effect of the former is negligible. Only cooperative strains with a very few available phenotypes are likely to be favored by selection. This undoubtedly inhibits the diversity of cooperative strains. Ensuing comes the parsimoniously low level of overall cooperation. On this front, cooperation coevolves with phenotypic diversity.

Our model integrates the subpopulation interactions with different phenotypes, and well captures the underlying rationale for many observations in the biological sphere. For instance, to surmount the competition from the commensal microbiota, *S*. *typhimurium* needs to express T1 to induce gut inflammation. The expression is costly, and the inflammation benefits the mutants as well. In direct competition, the cooperating *S*. *typhimurium* would absolutely get eliminated by the mutants (i.e., defectors). Real situation goes this way. Only a fraction of the cells in the population of *S*. *typhimurium* express T1, while the remaining do not. In a natural way, the expression can be regarded as conducting cooperative action. By virtue of this probabilistic expression of phenotype, the population of *S*. *typhimurium* outperforms the mutants [42]. Another relevant example may be the side-blotched male lizards found on the Pacific Coast of North America. These male lizards exhibit differing phenotypes in their throat color, either yellow or orange or blue [62, 63]. It is most probably that the phenomenon is the result of development rooted in our model philosophy. This conjecture has implications for and awaits the confirmation of field studies.

The impact of environmental variability on population survival in ecological systems has been intensely investigated, especially from the perspective of experimentation [34, 40, 41, 43]. In our model, due to the fact that individuals possess diverse potentially expressible phenotypes, there are multiple potential states for the population to inhabit due to random expression and inherent phenotype diversity. Cooperators, in competition with defectors, may have different viability as the population state varies. How cooperators fare when the population switches between these states is still unclear. We originally address this issue by combining phenotype-similarity based interaction and inherent phenotype diversity. Allowing individuals to vary in the number of phenotypes they can switch to provides many potential states for the population to reside. In some of these states, cooperative strains dominate the population, and the dynamics are stable in the sense that the population is less likely to be invaded by other strains. In other states, the dynamics manifest drastic oscillations; averagely speaking, cooperative strains prevail in the evolutionary competition with defective strains.

Experimental findings have established that the bet-hedging strategy (stochastic switching between phenotypes) can persist by its inherent adaptation to the fluctuating environmental conditions [34–38]. Our work generates qualitatively similar results that natural selection favors cooperation if contingent cooperators are able to switch to novel phenotypes at random, as well as that the population resides most frequently in the states exhibiting the optimal phenotypic diversity. Possessing diverse phenotypes while expressing one plays a similar role as the recombination of tag-trait does in Ref. [28], both loosening the coupling between tag and trait. In this sense, our mechanism can be added into ‘other mechanisms that can accomplish the same stabilizing effect’ as the authors of Ref. [28] have suggested.

The application of the framework we introduce here includes, but not limited to, the study of cooperation. It is a general theory for studying situations where in-group members play one game while out-group members play another game [64]. Using proper types of game to characterize in-group and out-group interactions, we can investigate and explain the evolution of parochial altruism and homophily [22], and corresponding results will be presented in another work. Applying this model to a variety of other social behaviors, such as coordination, trust and bargaining, may reveal more about such evolutionary dynamics and be worthy of further investigation [65–67], as is exploring situations where individuals are members of multiple groups with competing allegiances. Extending this framework to group interactions [68–70], which cannot always be viewed as the sum of pairwise interactions [71, 72], will be useful.

## Supporting information

S1 Supplementary Information Coevolutionary dynamics of phenotypic diversity and contingent cooperation. (PDF)
