# Supplementary material for: Coevolutionary dynamics of phenotypic diversity and contingent cooperation
Source: PLoS Comput Biol. 2017 Jan 31;13(1):e1005363. doi: 10.1371/journal.pcbi.1005363 (PMC5308777; doi:10.1371/journal.pcbi.1005363)
Supplement: S1 Supplementary Information — (PDF) [file pcbi.1005363.s001.pdf]

Supplementary Information: Coevolutionary dynamics of phenotypic diversity and contingent cooperation

Te Wu[1,2,◕], Long Wang[3*], Feng Fu[4,5,◕*]

1 Center for Complex Systems, Xidian University, Xi'an, China
2 Department of Applied Mathematics, The Hong Kong Polytechnic University, Hung Hom, Hong Kong, China
3 Center for Systems and Control, College of Engineering, Peking University, Beijing, China
4 Department of Mathematics, Dartmouth College, Hanover, United States of America
5 Department of Biomedical Data Science, Geisel School of Medicine, Dartmouth College, Hanover, United States of America

◕These authors contributed equally to this work.
* Email: fufeng@gmail.com (F.F.), longwang@pku.edu.cn (L.W.)

Simplified transition rates under strong selection

In the limit of strong selection (i.e., $\beta \to +\infty$), the formula for the transition rate as given in Eq. (1) can be greatly simplified. In terms of the strategy of residents and invading mutants, and of the number of potentially expressible phenotypes, there are ten cases. Denote by K_X and K_Y the numbers of potentially expressible phenotypes that the residents and the mutants can switch to, respectively. Denote by $f_X(i)$ and $g_Y(i)$ the fitness of each resident and each mutant respectively, when there are $N-i$ Xs and i Ys present in the population. The fixation probability that an invader Y successfully invades and replaces the otherwise population of Xs is $\phi(Y \to X) = [1 + \sum_{k=1}^{N-1} \prod_{i=1}^{k} \frac{f_X(i)}{g_Y(i)}]^{-1}$. We would like to point out that we include self interaction when calculating payoff. The inclusion has a remarkable effect on the fixation probability for the strong selection, while negligible for the weak selection.

Case 1: $X = D$ and $Y = D$, and $K_X < K_Y$.

Whenever X and Y express different phenotypes,

$$\begin{aligned} g_Y(i) &= e^{\beta(-\theta K_Y)}. \\ f_X(i) &= e^{\beta(-\theta K_X)}. \\ \frac{f_X(i)}{g_Y(i)} &= e^{\beta\theta(K_Y - K_X)}. \end{aligned}$$

For $K_X < K_Y$, it is obvious $\phi^d(Y \to X) = 0$.

Whenever X and Y express the same phenotype, following similar logic, we have $\phi^s(Y \to X) = 0$.

Therefore, $r(X, Y; K_X, K_Y) = 0$.

Case 2: $X = D$ and $Y = D$, and $K_X > K_Y$.

Similar to case 1, we can easily obtain $\phi^d(Y \to X) = 1$, and $\phi^s(Y \to X) = 1$.

Therefore, $r(X, Y; K_X, K_Y) = \frac{1}{2}$.

Case 3: $X = C$ and $Y = C$, and $K_X < K_Y$.

Whenever X and Y express different phenotypes,

$$\begin{aligned} g_Y(i) &= e^{\beta[i(b-c)-\theta K_Y]}. \\ f_X(i) &= e^{\beta[(b-c)(N-i)-\theta K_X]}. \\ \frac{f_X(i)}{g_Y(i)} &= e^{\beta[(b-c)(N-2i)-\theta(K_X-K_Y)]}. \\ \sum_{k=1}^{N-1}\prod_{i=1}^{k}\frac{f_X(i)}{g_Y(i)} &= \sum_{k=1}^{N-1} e^{\beta[\frac{k}{2}(N-2+N-2k)(b-c)-k\theta(K_X-K_Y)]} \\ &= \sum_{k=1}^{N-1} e^{\beta[k(N-1-k)(b-c)+k\theta(K_Y-K_X)]}. \end{aligned}$$

Notice that $k(N-1-k)(b-c)+k\theta(K_Y-K_X)>0$ always holds for $K_Y>K_X$ and $k\in\{1,2,...,N-1\}$, thus $\phi^d(Y\to X)=0$.

Whenever X and Y express the same phenotype,

$$\begin{aligned} g_Y(i) &= e^{\beta[N(b-c)-\theta K_Y]}. \\ f_X(i) &= e^{\beta[N(b-c)-\theta K_X]}. \\ \frac{f_X(i)}{g_Y(i)} &= e^{\beta\theta(K_Y-K_X)}. \end{aligned}$$

Thus, $\phi^s(Y\to X)=0$. Therefore, $r(X,Y;K_X,K_Y)=0$.

Case 4: $X=C$ and $Y=C$, and $K_X>K_Y$.

Whenever X and Y express different phenotypes, we still have

$$\begin{aligned} \sum_{k=1}^{N-1}\prod_{i=1}^{k}\frac{f_X(i)}{g_Y(i)} &= \sum_{k=1}^{N-1} e^{\beta[k(N-1-k)(b-c)+k\theta(K_Y-K_X)]} \\ &= \sum_{k=1}^{N-1} e^{\beta(b-c)[k(N-1-k)+\frac{k\theta(K_Y-K_X)}{b-c}]}. \end{aligned}$$

Let

$$\begin{aligned} A(k) &= (N-1-k)k-\frac{k\theta(K_X-K_Y)}{b-c} \\ &= -\left[k-\frac{1}{2}\left(N-1-\frac{\theta(K_X-K_Y)}{b-c}\right)\right]^2+\frac{1}{4}\left[N-1-\frac{\theta(K_X-K_Y)}{b-c}\right]^2. \end{aligned}$$

Obviously, we have $\frac{1}{2}\big(N-1-\frac{\theta(K_X-K_Y)}{b-c}\big)\le N-1$ for $K_X>K_Y$. The inequality $\frac{1}{2}\big(N-1-\frac{\theta(K_X-K_Y)}{b-c}\big)\ge 1$ necessitates $\theta\le\frac{(N-3)(b-c)}{K_X-K_Y}$. Under this condition, there exists a unique $k_0\in\{1,2,...,N-1\}$ and $\epsilon\in(-\frac{1}{2},\frac{1}{2})$ such that $\frac{1}{2}\big(N-1-\frac{\theta(K_X-K_Y)}{b-c}\big)=k_0+\epsilon$. So $A(k)$ achieves the maximal value at $k=k_0$ and $A(k_0)=k_0^2+2k_0\epsilon>0$ always holds. So $\phi^d(Y\to X)=0$. For $\theta>\frac{(N-3)(b-c)}{K_X-K_Y}$, $A(k)$ achieves the maximal value at $k=1$ as $A(1)=N-2-\frac{\theta(K_X-K_Y)}{b-c}$. Let $A(1)\ge 0$, we get $\theta\le\frac{(N-2)(b-c)}{K_X-K_Y}$. So

$$\phi^d(Y\to X)=\begin{cases} 0 & \text{if } \theta<\frac{(N-2)(b-c)}{K_X-K_Y}; \\ \frac{1}{2} & \text{if } \theta=\frac{(N-2)(b-c)}{K_X-K_Y}; \\ 1 & \text{if } \theta>\frac{(N-2)(b-c)}{K_X-K_Y}. \end{cases}$$

Whenever X and Y express the same phenotype, we have

$$\begin{aligned} g_Y(i) &= e^{\beta[N(b-c)-\theta K_Y]}. \\ f_X(i) &= e^{\beta[N(b-c)-\theta K_X]}. \\ \frac{f_X(i)}{g_Y(i)} &= e^{\beta\theta(K_Y-K_X)}. \end{aligned}$$

So, $\phi^s(Y \to X) = 1$ for $K_Y < K_X$.

Therefore,

$$r(X,Y;K_X,K_Y) = \begin{cases} \frac{1}{2K_X} & \text{if } \theta < \frac{(N-2)(b-c)}{K_X-K_Y}; \\ \frac{1}{4}(1+\frac{1}{K_X}) & \text{if } \theta = \frac{(N-2)(b-c)}{K_X-K_Y}; \\ \frac{1}{2} & \text{if } \theta > \frac{(N-2)(b-c)}{K_X-K_Y}. \end{cases}$$

Case 5: $X = C$ and $Y = D$, and $K_X < K_Y$.

Whenever X and Y express different phenotypes,

$$\begin{aligned} g_Y(i) &= e^{\beta(-\theta K_Y)}. \\ f_X(i) &= e^{\beta[(b-c)(N-i)-\theta K_X]}. \\ \frac{f_X(i)}{g_Y(i)} &= e^{\beta[(b-c)(N-i)+\theta(K_Y-K_X)]}. \end{aligned}$$

Since $(N-i)(b-c)+\theta(K_Y-K_X) > 0$ always holds for $K_Y > K_X$ and $i \in \{1,2,...,N-1\}$, so $\phi^d(Y \to X) = 0$.

Whenever X and Y express the same phenotype,

$$\begin{aligned} g_Y(i) &= e^{\beta[(N-i)b-\theta K_Y]}. \\ f_X(i) &= e^{\beta[(N-i)b-Nc-\theta K_X]}. \\ \frac{f_X(i)}{g_Y(i)} &= e^{\beta[-Nc+\theta(K_Y-K_X)]}. \end{aligned}$$

We get $\theta \geq \frac{Nc}{K_Y-K_X}$ by solving the inequality $-Nc+\theta(K_Y-K_X) \geq 0$. Thus,

$$\phi^s(Y \to X) = \begin{cases} 0 & \text{if } \theta > \frac{Nc}{K_Y-K_X}; \\ \frac{1}{N} & \text{if } \theta = \frac{Nc}{K_Y-K_X}; \\ 1 & \text{if } \theta < \frac{Nc}{K_Y-K_X}. \end{cases}$$

Therefore,

$$r(X,Y;K_X,K_Y) = \begin{cases} 0 & \text{if } \theta > \frac{Nc}{K_Y-K_X}; \\ \frac{1}{2NK_X} & \text{if } \theta = \frac{Nc}{K_Y-K_X}; \\ \frac{1}{2K_X} & \text{if } \theta < \frac{Nc}{K_Y-K_X}. \end{cases}$$

Case 6: $X = C$ and $Y = D$, and $K_X > K_Y$.

Whenever X and Y express different phenotypes,

$$\begin{aligned} g_Y(i) &= e^{\beta(-\theta K_Y)}. \\ f_X(i) &= e^{\beta[(N-i)(b-c)-\theta K_X]}. \\ \frac{f_X(i)}{g_Y(i)} &= e^{\beta[(N-i)(b-c)+\theta(K_Y-K_X)]}. \\ \sum_{k=1}^{N-1}\prod_{i=1}^{k}\frac{f_X(i)}{g_Y(i)} &= \sum_{k=1}^{N-1} e^{\beta[\frac{1}{2}k(2N-k-1)(b-c)+k\theta(K_Y-K_X)]} \\ &= \sum_{k=1}^{N-1} e^{\beta(b-c)[k(N-\frac{k}{2}-\frac{1}{2})-\frac{\theta k(K_X-K_Y)}{b-c}]}. \end{aligned}$$

Let

$$
\begin{aligned}
A(k) &= k(N-\frac{k}{2}-\frac{1}{2})-\frac{\theta k(K_X-K_Y)}{b-c}\\
&= -\frac{1}{2}\left[k-\left(N-\frac{1}{2}-\frac{\theta(K_X-K_Y)}{b-c}\right)\right]^2+\frac{1}{4}\left[N-\frac{1}{2}-\frac{\theta(K_X-K_Y)}{b-c}\right]^2.
\end{aligned}
$$

We obtain $\frac{b-c}{2(K_X-K_Y)}\le\theta\le\frac{(2N-3)(b-c)}{2(K_X-K_Y)}$ by solving the set of inequalities $1\le N-\frac{1}{2}-\frac{\theta(K_X-K_Y)}{b-c}\le N-1$. At this time, $\phi^d(Y\to X)=0$. For $\theta>\frac{(2N-3)(b-c)}{2(K_X-K_Y)}$, $A(k)$ achieves the maximal value at $k=1$ and $A(1)=N-1-\frac{\theta(K_X-K_Y)}{b-c}$. The inequality $A(1)\ge 0$ requires $\theta\le\frac{(N-1)(b-c)}{K_X-K_Y}$. For $\theta<\frac{b-c}{2(K_X-K_Y)}$, $A(k)$ achieves the maximal value at $k=N-1$ and $A(N-1)=\frac{N(N-1)}{2}-\frac{\theta(N-1)(K_X-K_Y)}{b-c}$. Let $A(N-1)\ge 0$, we get $\theta\le\frac{N(b-c)}{2(K_X-K_Y)}$. So

$$
\phi^d(Y\to X)=\begin{cases}0 & \text{if }\ \theta<\frac{(N-1)(b-c)}{K_X-K_Y};\\ \frac{1}{2} & \text{if }\ \theta=\frac{(N-1)(b-c)}{K_X-K_Y};\\ 1 & \text{if }\ \theta>\frac{(N-1)(b-c)}{K_X-K_Y}.\end{cases}
$$

Whenever X and Y express the same phenotype,

$$
\begin{aligned}
g_Y(i) &= e^{\beta[(N-i)b-\theta K_Y]}.\\
f_X(i) &= e^{\beta[(N-i)b-Nc-\theta K_X]}.\\
\frac{f_X(i)}{g_Y(i)} &= e^{\beta[-Nc-\theta(K_X-K_Y)]}.
\end{aligned}
$$

Since $-Nc-\theta(K_X-K_Y)<0$ always holds for $K_Y<K_X$, so $\phi^s(Y\to X)=1$.

Therefore,

$$
r(X,Y;K_X,K_Y)=\begin{cases}\frac{1}{2K_X} & \text{if }\ \theta<\frac{(N-1)(b-c)}{K_X-K_Y};\\ \frac{1}{4}(1+\frac{1}{K_X}) & \text{if }\ \theta=\frac{(N-1)(b-c)}{K_X-K_Y};\\ \frac{1}{2} & \text{if }\ \theta>\frac{(N-1)(b-c)}{K_X-K_Y}.\end{cases}
$$

Case 7: $X=D$ and $Y=C$, and $K_X<K_Y$.

Whenever X and Y express different phenotypes,

$$
\begin{aligned}
g_Y(i) &= e^{\beta[(b-c)i-\theta K_Y]}.\\
f_X(i) &= e^{\beta(-\theta K_X)}.\\
\frac{f_X(i)}{g_Y(i)} &= e^{\beta[-(b-c)i+\theta(K_Y-K_X)]}.\\
\sum_{k=1}^{N-1}\prod_{i=1}^{k}\frac{f_X(i)}{g_Y(i)} &= \sum_{k=1}^{N-1}e^{\frac{1}{2}\beta(b-c)[-k(k+1)+\frac{2k\theta(K_Y-K_X)}{b-c}]}.
\end{aligned}
$$

Let

$$
\begin{aligned}
A(k) &= -k(k+1)+\frac{2k\theta(K_Y-K_X)}{b-c}\\
&= -\left[k+\frac{1}{2}\left(1-\frac{2\theta(K_Y-K_X)}{b-c}\right)\right]^2+\frac{1}{4}\left[1-\frac{2\theta(K_Y-K_X)}{b-c}\right]^2.
\end{aligned}
$$

We obtain $\theta \leq \frac{(2N-1)(b-c)}{2(K_Y-K_X)}$ and $\theta \geq \frac{3(b-c)}{2(K_Y-K_X)}$ by solving the inequality $-\frac{1}{2}(1-\frac{2\theta(K_Y-K_X)}{b-c}) \leq N-1$ and $-\frac{1}{2}(1-\frac{2\theta(K_Y-K_X)}{b-c}) \geq 1$, respectively. Whenever $\theta > \frac{(2N-1)(b-c)}{2(K_Y-K_X)}$, $A(k)$ achieves the maximal value at $k = N-1$ and $A(N-1) = -(N-1)\big[N - \frac{2\theta(K_Y-K_X)}{b-c}\big]$. It can be easily verified that $A(N-1) > 0$ always holds for $\theta > \frac{(2N-1)(b-c)}{2(K_Y-K_X)}$. In another situation $\theta < \frac{3(b-c)}{2(K_Y-K_X)}$, $A(k)$ achieves the maximal value at $k = 1$ and $A(1) = -2 + \frac{2\theta(K_Y-K_X)}{b-c}$. Let $A(1) \geq 0$, we get $\theta \geq \frac{b-c}{K_Y-K_X}$. Therefore, we have

$$\phi^d(Y \to X) = \begin{cases} 0 & \text{if } \theta > \frac{(b-c)}{K_Y-K_X}; \\ \frac{1}{2} & \text{if } \theta = \frac{(b-c)}{K_Y-K_X}; \\ 1 & \text{if } \theta < \frac{(b-c)}{K_Y-K_X}. \end{cases}$$

Whenever X and Y express the same phenotype,

$$\begin{aligned} g_Y(i) &= e^{\beta[ib-Nc-\theta K_Y]}. \\ f_X(i) &= e^{\beta(ib-\theta K_X)}. \\ \frac{f_X(i)}{g_Y(i)} &= e^{\beta[Nc+\theta(K_Y-K_X)]}. \end{aligned}$$

As $Nc + \theta(K_Y - K_X) > 0$ always holds for $K_Y > K_X$, we have $\phi^s(Y \to X) = 0$.

Therefore, we have

$$r(X, Y; K_X, K_Y) = \begin{cases} 0 & \text{if } \theta > \frac{(b-c)}{K_Y-K_X}; \\ \frac{1}{4}(1-\frac{1}{K_X}) & \text{if } \theta = \frac{(b-c)}{K_Y-K_X}; \\ \frac{1}{2}(1-\frac{1}{K_X}) & \text{if } \theta < \frac{(b-c)}{K_Y-K_X}. \end{cases}$$

Case 8: $X = D$ and $Y = C$, and $K_X > K_Y$.

Whenever X and Y express different phenotypes,

$$\begin{aligned} g_Y(i) &= e^{\beta[i(b-c)-\theta K_Y]}. \\ f_X(i) &= e^{\beta(-\theta K_X)}. \\ \frac{f_X(i)}{g_Y(i)} &= e^{\beta[-i(b-c)-\theta(K_X-K_Y)]}. \end{aligned}$$

Since $-i(b-c) - \theta(K_X - K_Y) < 0$ always holds for $K_X > K_Y$ and $i \in \{1, 2, ..., N-1\}$, we have $\phi^d(Y \to X) = 1$.

Whenever X and Y express the same phenotype,

$$\begin{aligned} g_Y(i) &= e^{\beta(ib-Nc-\theta K_Y)}. \\ f_X(i) &= e^{\beta(ib-\theta K_X)}. \\ \frac{f_X(i)}{g_Y(i)} &= e^{\beta[Nc+\theta(K_Y-K_X)]}. \end{aligned}$$

The inequality $Nc + \theta(K_Y - K_X) \geq 0$ implies $\theta \leq \frac{Nc}{K_X-K_Y}$. So we have

$$\phi^s(Y \to X) = \begin{cases} 0 & \text{if } \theta < \frac{Nc}{K_X-K_Y}; \\ \frac{1}{N} & \text{if } \theta = \frac{Nc}{K_X-K_Y}; \\ 1 & \text{if } \theta > \frac{Nc}{K_X-K_Y}. \end{cases}$$

Therefore,

$$r(X,Y;K_X,K_Y) = \begin{cases} \frac{1}{2}(1-\frac{1}{K_X}) & \text{if } \theta < \frac{Nc}{K_X-K_Y}; \\ \frac{1}{2}-(1-\frac{1}{N})\frac{1}{2K_X} & \text{if } \theta = \frac{Nc}{K_X-K_Y}; \\ \frac{1}{2} & \text{if } \theta > \frac{Nc}{K_X-K_Y}. \end{cases}$$

Case 9: $X = D$ and $Y = C$, and $K_X = K_Y$.

Whenever X and Y express different phenotypes,

$$\begin{aligned} g_Y(i) &= e^{\beta[i(b-c)-\theta K_Y]}. \\ f_X(i) &= e^{\beta(-\theta K_X)}. \\ \frac{f_X(i)}{g_Y(i)} &= e^{-\beta(b-c)i}. \\ \sum_{k=1}^{N-1}\prod_{i=1}^{k}\frac{f_X(i)}{g_Y(i)} &= \sum_{k=1}^{N-1} e^{-\frac{1}{2}\beta k(k+1)(b-c)}. \end{aligned}$$

Thus, $\phi^d(Y \to X) = 1$.

Whenever X and Y express the same phenotype,

$$\begin{aligned} g_Y(i) &= e^{\beta(ib-Nc-\theta K_Y)}. \\ f_X(i) &= e^{\beta(ib-\theta K_X)}. \\ \frac{f_X(i)}{g_Y(i)} &= e^{\beta Nc}. \end{aligned}$$

Since $Nc > 0$ always holds, $\phi^s(Y \to X) = 0$.

Therefore, $r(X,Y;K_X,K_Y) = \frac{1}{2}(1-\frac{1}{K_X})$.

Case 10: $X = C$ and $Y = D$, and $K_X = K_Y$.

Whenever X and Y express different phenotypes,

$$\begin{aligned} g_Y(i) &= e^{\beta(-\theta K_Y)}. \\ f_X(i) &= e^{\beta[(N-i)(b-c)-\theta K_X]}. \\ \frac{f_X(i)}{g_Y(i)} &= e^{\beta(b-c)(N-i)}. \\ \sum_{k=1}^{N-1}\prod_{i=1}^{k}\frac{f_X(i)}{g_Y(i)} &= \sum_{k=1}^{N-1} e^{\beta k(N-\frac{k}{2}-\frac{1}{2})(b-c)}. \end{aligned}$$

For $k \in \{1,2,...,N-1\}$, we have $k(N-\frac{k}{2}-\frac{1}{2})(b-c) > 0$. So $\phi^d(Y \to X) = 0$.

Whenever X and Y express the same phenotype,

$$\begin{aligned} g_Y(i) &= e^{\beta[(N-i)b-\theta K_Y]}. \\ f_X(i) &= e^{\beta[(N-i)b-Nc-\theta K_X]}. \\ \frac{f_X(i)}{g_Y(i)} &= e^{\beta(-Nc)}. \end{aligned}$$

Since $-Nc < 0$ always holds, $\phi^s(Y \to X) = 1$.

Therefore, $r(X,Y;K_X,K_Y) = \frac{1}{2K_X}$.
